# Supplementary material for: Real-World Treatment Outcomes and Safety of Afatinib in Advanced Squamous Cell Lung Cancer Progressed after Platinum-Based Doublet Chemotherapy and Immunotherapy (SPACE Study)
Source: Cancers (Basel). 2023 Nov 24;15(23):5568. doi: 10.3390/cancers15235568 (PMC10705085; doi:10.3390/cancers15235568)
Supplement: Supplementary file 1 [file cancers-15-05568-s001.zip › cancers-2669722-supplementary.pdf]

Supplementary Tables

Table S1. List of the 51 actionable genes in the Cancer-PRIME^{TM} next-generation sequencing panel.

ABL1	BTK	ERBB3	GNA11	MET	PPARG	TP53
AKT1	CCND1	ERBB4	GNAQ	MTOR	PTCH1	TSC1
ALK	CDK4	ESR1	HRAS	MYCN	PTEN	TSC2
AR	CDK6	FGFR1	IDH1	NOTCH1	RAF1	
BAP1	CDKN2A	FGFR2	IDH2	NRAS	RET	
BRAF	DDR2	FGFR3	KIT	PDGFRA	ROS1	
BRCA1	EGFR	FGFR4	KRAS	PD-L1	SMO	
BRCA2	ERBB2	FLT3	MAP2K1	PIK3CA	STK11	

Table S2. Disease control rate (DCR) according to different clinicopathologic parameters in response evaluable patients (n=37)

	DCR	p-value	Univariate	
			OR (95% CI)	p-value
All	59.5			
Age		0.549		
<70	55.0		reference	
≥70	64.7		1.50 (0.39-5.66)	0.550
Sex		0.633		
Male	57.6		reference	
Female	75.0		2.21 (0.21-23.56)	0.511
Smoking History		>0.999		
Never	50.0		reference	
Ever	60.0		1.50 (0.09-26.01)	0.781
Smoking intensity		0.708		
<30 pack-years	50.0		reference	
≥30 pack-years	63.0		1.70 (0.39-7.36)	0.478
ECOG PS		0.711		
0, 1	57.1		Reference	

≥2	66.7		1.50 (0.31-7.25)	0.614
Stage*		0.465		
IVA	64.0		2.13 (0.51-9.01)	0.303
IVB	45.5		reference	
Differentiation		0.356		
Well/moderate	70.6		3.20 (0.52-19.84)	0.212
Poor/unknown	42.9		reference	
No. of previous lines of systemic therapy before afatinib		0.488		0.708
1-3	64.0		1.78 (0.44-7.17)	
≥4	50.0		reference	
Type of previous systemic therapy†		>0.999		0.771
Chemoimmunotherapy	50.0		reference	
Platinum-doublet followed by immunotherapy	60.7		1.55 (0.19-12.64)	0.894
Involved organ		0.742		
<3	57.1		reference	
≥3	62.5		1.25 (0.33-4.73)	0.743
Brain metastasis		0.408		
No	45.0		2.30 (0.43-12.25)	0.328
Yes	42.9		reference	
Liver metastasis		0.228		
No	65.5		3.17 (0.63-16.05)	0.164
Yes	37.5		reference	
PD-L1 TPS‡		0.733		0.735
<1%	70.0		1.46 (0.25-8.43)	0.502
1-49%	53.8		0.73 (0.15-3.47)	0.480
≥50%	61.5		reference	
Afatinib starting dose		>0.999		
30mg	60.0		1.03 (0.24-4.53)	0.968
40mg	59.3		reference	
Dose modification				0.099
No	47.6	0.176	reference	
Yes	75.0		3.30 (0.79-13.64)	

Multivariate analysis was not necessary because there was only one variable with a p-value < 0.1.

*After excluding 1 patient with stage IIIC

†After excluding 1 patient who received immunotherapy followed by platinum-doublet

‡After excluding 1 patient without data on PD-L1 expression

OR, odds ratio; CI, confidence interval; ECOG PS, Eastern Cooperative Oncology Group performance status; PD-L1, programmed death ligand 1; TPS, tumor proportion score.

Table S3. TTF analyses results according to the clinicopathological parameters of all study subjects (n=42)

	Median TTF (months)	Univariate	
		HR (95% CI)	p-value
All	2.1		
Age			0.576
<70	2.0	reference	
≥70	2.1	1.19 (0.64-2.25)	
Sex			0.236
Male	1.9	1.89 (0.66-5.45)	
Female	4.7	reference	
Smoking history			0.599
Never	2.1	1.47 (0.35-6.23)	
Ever	2.1	reference	
Smoking intensity			0.684
<30 pack-years	1.5	reference	
≥30 pack-years	2.1	1.16 (0.56-2.42)	
ECOG PS			0.286
0, 1	2.1	1.52 (0.71-3.25)	
≥2	2.8	reference	
Stage*			0.196
IVA	2.3	reference	
IVB	1.8	1.59 (0.79-2.18)	
Differentiation			0.474
Well to moderate	2.7	reference	
Poor or unknown,	1.5	1.39 (0.56-3.48)	
No. of previous systemic therapy before afatinib			0.920

1-3	2.1	1.28 (0.62-2.41)	
≥4	2.1	reference	
Types of previous systemic therapy†			0.187
Chemoimmunotherapy	1.6	1.74 (0.52-5.88)	
Platinum-doublet followed by immunotherapy	2.1	0.74 (0.28-1.96)	
Involved organ			0.762
<3	2.1	1.10 (0.58-2.08)	
≥3	2.1	reference	
Brain metastasis			0.420
No	2.1	reference	
Yes	1.3	1.38 (0.63-3.03)	
Liver metastasis			0.179
No	2.3	reference	
Yes	1.8	1.76 (0.77-4.02)	
PD-L1 TPS‡			0.439
<1%	1.8	reference	
1-49%	2.0	1.32 (0.59-2.92)	
≥50%	2.8	0.79 (0.35-1.75)	
Afatinib starting dose			0.892
30mg	1.9	reference	
40mg	2.1	1.05 (0.49-2.23)	
Dose modification			0.029
No	1.5	2.07 (1.08-3.97)	
Yes	3.0	reference	

Multivariate analysis was not necessary because there was only one variable with a p-value < 0.1.

*After excluding 1 patient with stage IIIC

†After excluding 1 patient who received immunotherapy followed by platinum-doublet

‡After excluding 1 patient without data on PD-L1 expression

TTF, time to treatment failure; HR, hazard ratio; CI, confidence interval; ECOG PS, Eastern Cooperative Oncology Group performance status; PD-L1, programmed death-ligand 1; TPS, tumor proportion score

Table S4. Overall survival analyses results according to clinicopathological parameters of all study subjects (n=42)

	Median OS (months)	Univariate	
		HR (95% CI)	p-value
All	6.1		
Age			0.406
<70	7.3	reference	
≥70	5.0	1.37 (0.66-2.85)	
Sex			0.374
Male	6.1	reference	
Female	7.1	1.082	
Smoking History			0.435
Never	NR	reference	
Ever	6.1	2.23 (0.29-16.67)	
Smoking intensity			0.722
<30 pack-years	6.5	1.15 (0.52-2.54)	
≥30 pack-years	6.1	reference	
ECOG PS			0.341
0, 1	6.1	reference	
≥2	3.4	1.52 (0.64-3.60)	
Stage*			0.829
IVA	7.3	reference	
IVB	6.1	1.11 (0.43-2.76)	
Differentiation			0.468
Well to moderate	5.0	1.46 (0.53-4.07)	
Poor/unknown	10.3	reference	
No. of previous lines of systemic therapy before afatinib			0.837
1-3	6.1	1.09 (0.49-2.39)	
≥4	6.0	reference	
Type of previous systemic therapy†			0.801
Chemoimmunotherapy	2.7	1.77 (0.29-10.67)	
Platinum-doublet followed by immunotherapy	6.8	reference	
Involved organ			0.435
<3	6.8	reference	
≥3	5.0	1.34 (0.64-2.78)	
Brain metastasis			0.472
No	6.8	reference	
Yes	5.0	1.43 (0.54-3.77)	
Liver metastasis			0.388
No	7.3	reference	
Yes	4.7	1.46 (0.62-3.45)	
PD-L1 TPS‡			0.320
<1%	2.8	reference	
1-49%	5.0	1.14 (0.45-2.90)	
≥50%	10.0	0.59 (0.24-1.46)	
Afatinib starting dose			0.073
30mg	3.7	2.11 (0.93-4.77)	

40mg	7.3	reference	
Dose modification			0.262
No	5.0	1.54 (0.72-3.30)	
Yes	6.1	reference	

Multivariate analysis was not necessary because there was only one variable with a p-value < 0.1.

*After excluding 1 patient with stage IIIC

†After excluding 1 patient who received immunotherapy followed by platinum-doublet

‡After excluding 1 patient without data on PD-L1 expression

OS, overall survival; HR, hazard ratio; CI, confidence interval; ECOG PS, Eastern Cooperative Oncology Group performance status; PD-L1, programmed death ligand 1; TPS, tumor proportion score.
